# Osmotic stress as a factor for regulating *E. coli* hydrogenase activity and enhancing H_2_ production during mixed carbon sources fermentation

**DOI:** 10.3934/microbiol.2023037

**Published:** 2023-11-06

**Authors:** Anush Babayan, Anait Vassilian, Karen Trchounian

**Affiliations:** 1 Department of Biochemistry, Microbiology and Biotechnology, Faculty of Biology, Yerevan State University, 0025 Yerevan, Armenia; 2 Scientific-Research Institute of Biology, Faculty of Biology, Yerevan State University, 0025 Yerevan, Armenia; 3 Microbial Biotechnologies and Biofuel Innovation Center, Yerevan State University, 0025 Yerevan, Armenia; 4 Department of Ecology and Nature Protection, Faculty of Biology, Yerevan State University, 0025 Yerevan, Armenia

**Keywords:** *Escherichia coli*, mixed carbon fermentation, Hyd enzymes, osmotic stress, pH

## Abstract

*Escherichia coli* performs mixed-acid fermentation and produces molecular hydrogen (H_2_) via reversible hydrogenases (Hyd). H_2_ producing activity was investigated during hyper- and hypo-osmotic stress conditions when a mixture of carbon sources (glucose and glycerol) was fermented at different pHs. Hyper-osmotic stress decreased H_2_ production rate (V_H2_) ~30 % in wild type at pH 7.5 when glucose was supplemented, while addition of formate stimulated V_H2_ ~45% compared to hypo-stress conditions. Only in *hyfG* in formate assays was V_H2_ inhibited ~25% compared to hypo-stress conditions. In hypo-stress conditions addition of glycerol increased V_H2_ ~2 and 3 fold in *hybC* and *hyfG* mutants, respectively, compared to wild type. At pH 6.5 hyper-osmotic stress stimulated V_H2_ ~2 fold in all strains except *hyaB* mutant when glucose was supplemented, while in formate assays significant stimulation (~3 fold) was determined in *hybC* mutant. At pH 5.5 hyper-osmotic stress inhibited V_H2_ ~30% in wild type when glucose was supplemented, but in formate assays it was stimulated in all strains except *hyfG*. Taken together, it can be concluded that, depending on external pH and absence of Hyd enzymes in stationary-phase-grown osmotically stressed *E. coli* cells, H_2_ production can be stimulated significantly which can be applied in developing H_2_ production biotechnology.

## 1. Introduction

*E. coli* is able to ferment various sole carbon sources such as sugars (glucose, xylose, galactose, etc.), alcohols (glycerol) or their mixtures [1]–[3]. During fermentation, different end products are generated. Among them, H_2_ gas is produced. H_2_ has a big potential to become one of the alternative energy sources that can be added to the current energy system, fulfilling the energy demands of the global market [4],[5]. H_2_ is an “eco-friendly” fuel that generates no toxic compounds, and only water is formed when H_2_ is burned.

H_2_ can be produced via different methods, but biological ones are considered future oriented and most promising [6]. Recently, by applying artificial microbial consortia, it was possible to surpass the “Thauer limit” of H_2_ yield (4 moles of H_2_ per mole of glucose) [7]. H_2_ is produced via four reversible membrane bound [Ni-Fe] hydrogenase (Hyd) enzymes. Hyd-1 and Hyd-2 are encoded by *hya* and *hyb* operons, respectively. Hyd-1 and Hyd-2 can work either in oxidizing or in producing mode depending on external pH and carbon source [8]. Hyd-3 encoded by *hyc* operon with formate dehydrogenase H (FDH-H) form formate hydrogen lyase (FHL-1) complex while Hyd-4 encoded by *hyf* operon forms FHL-2 complex [9]–[11]. During glucose fermentation, Hyd-3 is a major H_2_ producing Hyd enzyme, while Hyd-4 is mainly responsible for H_2_ uptake or, at pH 5.5 together with Hyd-3, forms a newly suggested H_2_ producing Hyd complex [12],[13]. When glycerol is fermented at pH 7.5, Hyd-2 mainly and Hyd-1 partially are responsible for H_2_ production.

Previously, it was shown that H_2_ production by *E. coli* is inhibited by *N,N′*-dicyclohexylcarbodiimide (DCCD) [8],[14], a specific inhibitor of the F_O_F_1_-ATPase, or disturbed in *atp* mutant (DK8 lacking F_O_F_1_) [15]. A relationship between F_O_F_1_ and Hyd enzymes has been shown in the literature and in different environmental conditions [16]–[18].

During sole glucose fermentation, H_2_ production has been determined to be sensitive to hyper-osmotic (hyper) and hypo-osmotic (hypo) stress at slightly alkaline pH [19]. However, this effect was eliminated when exogenous formate was added. This was the first indication of osmotic sensitivity of Hyd enzymes and could be related to their operation mode. Moreover, Hyd-4 is suggested to be sensitive to osmotic stress during sole glucose fermentation [8],[16]. In addition, during sole glycerol fermentation, it was shown that besides Hyd-4, Hyd-3 is also osmosensitive but at different pH. Moreover, during glycerol fermentation, osmosensitivity of Hyd-4 was established for pH 6.5 [19].

Cell osmoregulation in bacteria is a complex phenomenon and needs thorough investigation. It is known that *E. coli* responds to osmotic stress by regulating K^+^ transport via TrkA system [20]–[22]. The latter forms a supercomplex with F_O_F_1_ during sole sugar fermentation, which might suggest that F_O_F_1_-Trk complex has an osmoregulatory function in the membrane [17]. However, many aspects of cell osmoregulation, especially metabolic cross talk of membrane bound proteins for maintaining cell turgor, are still complex problems which need deeper investigation.

Cell turgor is the hydrostatic pressure difference that balances the difference in internal and external osmolyte concentration [23]. Due to the small size of bacteria, turgor is experimentally quite difficult to determine, and with the use of different techniques, values for the magnitude of turgor in *Escherichia coli* differing by a factor of ten have been reported; values range between 30 kPa (0.3 atm) (42) and 300 kPa (3 atm). The considerably higher turgor pressure measured for *Bacillus subtilis* (1.9 MPa) (19 atm) is generally thought to be required to stretch the much thicker peptidoglycan sacculus of this Gram-positive bacterium when the cell doubles its volume before it divides [24]. Turgor is generally considered essential for growth [25],[26], but there is still considerable debate as to whether turgor presses the cytoplasmic membrane onto the peptidoglycan sacculus or the cytoplasm and periplasm of Gram-negative bacteria are actually isosmotic, which would make the outer membrane the turgor-restraining cellular structure [25]. No microorganism can actively pump water into or out of the cytoplasm to compensate for the osmotically instigated water fluxes across the cytoplasmic membrane. Hence, cellular adjustments to both hyper- and hypoosmotic stress must rely on indirect countermeasures that allow the cell to direct and scale water influxes or effluxes as the environmental osmolality fluctuates [23].

In the current study, the role of Hyd enzymes in H_2_ production during osmotic stress conditions when mixed carbon sources (glucose and glycerol) were fermented was investigated. Osmotic stress as a factor for regulation of H_2_ metabolism has been suggested.

## 2. Materials and methods

### 2.1. Bacterial strains and cultivation

The characteristics of the *E. coli* strains used in the study are described in Table 1. Bacterial cultures were grown overnight under anaerobic fermentative conditions and transferred into high buffered growth medium containing peptone (20 g L^−1^) at pH of 7.5, 6.5 or 5.5, with salt compositions as follows: 15 g L^−1^ K_2_HPO_4_, 1.08 g L^−1^ KH_2_PO_4_ and 5 g L^−1^ NaCl (pH 7.5); 7.4 g L^−1^ K_2_HPO_4_, 8.6 g L^−1^ KH_2_PO_4_ and 5 g L^−1^ NaCl (pH 6.5); and 1.08 g L^−1^ K_2_HPO_4_, 15 g L^−1^ KH_2_PO_4_ and 5 g L^−1^ NaCl (pH 5.5). The medium was supplemented with 2 g L^−1^ glucose and 10 g L^−1^ glycerol. Bacterial overnight cultures were carried out in the same way as for the buffered growth medium for each pH and supplement added [12],[19].

**Table 1.** Characteristics of *E. coli* wild type and mutant strains used.

Strains	Genotype	Reference
Wild type	BW25113 *rrnB ΔlacZ4787HsdR514Δ(araBAD)567 Δ(rhaBAD)568 rph-1*	[27]
JW0955 Km^R*^	BW 25113 *ΔhyaB*	[27]
JW2962 Km^R*^	BW 25113 *ΔhybC*	[27]
JW2691 Km^R*^	BW 25113 *ΔhycE*	[27]
JW2472 Km^R*^	BW25113 *ΔhyfG*	[27]

*Resistant to kanamycin

Bacterial cultures were grown in sealed flasks under fermentative conditions for 18–24 h at 37 °C; anaerobic conditions in the medium were achieved by displacing O_2_ during autoclaving [16],[24]. The medium pH was determined using a pH meter with selective pH electrode (HJ1131B, Hanna Instruments, Portugal) and adjusted to the required values (see above) with 0.1 M NaOH or 0.1 N HCl.

### 2.2. Redox potential determination and hydrogen production assays

Redox potential (E_h_) in bacterial biomass was determined using two different redox electrodes: a titanium-silicate (Ti-Si) one (EO-02, Gomel State Enterprise of Electrometric Equipment (GSEEE), Gomel, Belarus) and a platinum (Pt) (EPB-1, GSEEE, or PT42BNC, Hanna Instruments, Portugal) glass electrode [12],[19],[28]. The Ti-Si electrode measures the overall E_h_, whereas the Pt electrode is responsive to H_2_ under anaerobic conditions [29]. The dual feature of the electrode system (Ti-Si/Pt) has been used [12],[19],[28] to detect H_2_ gas production in bacterial biomass by measuring the H_2_ production rate (V_H2_) of bacteria. The latter is calculated as the difference between the initial rates of decrease in the Pt and Ti-Si electrodes' readings per min and expressed in mV of E_h_ per min per mg cell dry weight (CDW).

This electrochemical approach applied for hydrogen determination is similar to the Clark-type electrode used by Fernandez [30] and other researchers [31]–[33]. As a control experiment, cells were used without any addition of carbon source. In this case, H_2_ production was absent. Importantly, the salt content of the solution did not affect the evolution of E_h_ by H_2_ saturation, and, moreover, supplementation of H_2_ into the solution did not have any impact on external or medium pH [34].

The cells were harvested, washed and transferred into assay medium (150 mM Tris-phosphate, at the indicated pH, containing of 0.4 mM MgSO_4_, 1 mM NaCl and 1 mM KCl) prior to the E_h_ measurements. When cells were washed in distilled water and transferred into the assay medium, bacteria were subjected to a hypo-stress whereas transfer from the other washing solution (0.8 M sucrose) into the assay medium was a hyper-stress [19],[22]. This approach was employed to study osmotic stress response by *E. coli*. The E_h_ measurements were performed in the assay buffer solution in a thermostatic chamber at a constant temperature of 37 °C to determine H_2_ production upon addition of 2 g L^−1^ glucose or 10 g L^−1^ glycerol or 0.68 g L^−1^ formate [35]. For the DCCD inhibition studies, the cells were incubated with the reagent at 0.2 mM.

### 2.3. Chemicals and data analysis and statistics

All reagents and chemicals used for experiments were of analytical grade (Sigma Aldrich, Carl Roth GmbH, Germany). The cell dry weight (CDW) was determined as described previously [16].

Average data obtained from three independent cell cultures are represented, and standard deviations of values do not exceed 3% if not given. Results are presented as mean ± SD. A p-value of less than 0.05 was considered significant. Data were visualized using GraphPad Prism 8 software. Significance (p < 0.05) was determined by two-way ANOVA and Tukey's multiple comparisons test. The comparisons of parameter values have been performed between wild type and mutant strain values in each condition.

## 3. Results and discussion

### 3.1. H_2_ production by E. coli wild type and mutant strains during hyper- and hypo-osmotic stress and inhibition by DCCD at pH 7.5

It is known that *E. coli* can utilize mixed carbon sources and produce various fermentation end products. The responsible Hyd enzymes have been detected during glucose and/or glycerol fermentation, and the role of proton ATPase has been evidenced before [8]. Osmotic stress was detected to affect Hyd enzymes at pH 7.5 during sole glucose or glycerol fermentative conditions [17],[19]. Nevertheless, during mixed carbon sources fermentation, the role of Hyd enzymes in relationship with proton ATPase under hypo-osmotic stress conditions is not investigated.

**Figure 1.**
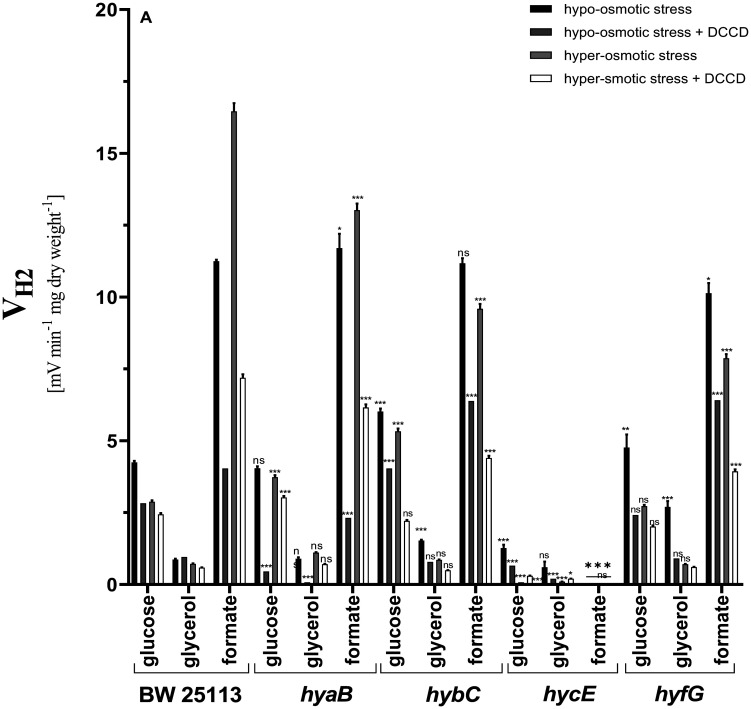
H_2_ production rates (V_H2_) by *E. coli* BW25113 wild type and different mutants with defects in Hyd enzymes under hypo- and hyper-osmotic stress during mixed carbon sources fermentation at pH 7.5. In the assays, glucose, glycerol or formate has been added in the concentrations as in growth medium. DCCD (0.2 mM) was added into the assay medium when indicated. For strains, see Table 1. Significance (p < 0.05) was determined by Tukey's multiple comparison test. Data are represented as mean ± SD. *p < 0.05, **p < 0.01, ***p < 0.001, ns – not significant, n = 3. For the others, see Materials and methods.

*E. coli* wild type cells grown on a mixture of glucose and glycerol in glucose assays reached an H_2_ production rate (V_H2_) of 4.25 mV E_h_/min/mg CDW. When in the assays formate was added, V_H2_ reached 11.25 mV E_h_/min/mg CDW (Figure 1). DCCD inhibited H_2_ production by 35% and 65% in glucose and formate assays, respectively. When glycerol was added into the assays, V_H2_ was similar with DCCD assays (Figure 1). During hyper-osmotic stress conditions in glucose assays, V_H2_ decreased ~35%, which was similar to the conditions with DCCD assays under hypo-osmotic conditions. This data suggests that the role of proton ATPase in H_2_ production and its regulation is significant. The obtained results confirm that proton ATPase is osmosensitive, which was detected earlier for sole glucose fermentative conditions in bacteria and plants [22],[35].

Interestingly, in formate assays hyper-osmotic stress stimulated H_2_ production by 40% (Figure 1). This phenomenon might be because the volume of periplasmatic space of the cells might be changed, which could affect the H^+^ transport and further H_2_ production via Hyd enzymes, which are membrane associated, and changing conformation could lead to enhanced H_2_ production. The main H_2_ producing enzyme during glucose fermentation is Hyd-3 [10],[13], and in formate assays Hyd-3 produces H_2_, while DCCD assays had similar effects with and without osmotic stress conditions. This confirms previously obtained data that at pH 7.5, during sole glucose fermentative conditions, Hyd-4 is osmosensitive [8],[17].

In glucose assays in hypo-osmotic stress conditions, in Hyd-2 mutant but not Hyd-1 mutant, V_H2_ was ~40% higher compared to wild type. Under hyper-osmotic stress conditions, V_H2_ was ~30% and ~85% higher in Hyd-1 and Hyd-2 mutants, respectively, compared to wild type. Moreover, in Hyd-1 mutant in DCCD assays during hypo-osmotic stress conditions, total inhibition of H_2_ production was determined, which was not detected in wild type cells. In addition, under hyper-osmotic stress conditions, DCCD inhibited V_H2_ ~20%, while in wild type cells no inhibition was shown (Figure 1). This might be because Hyd-1 and proton ATPase are interacting to balance the transmembrane proton gradient and thus proton motive force, and absence of Hyd-1 is compensated by enhanced activity of proton ATPase for transporting protons out of the cell. A similar idea about the relationship between F_O_F_1_ and Hyd enzymes has been shown but in other environmental conditions [2], [8], [18]. Alternatively, it might be suggested that Hyd-1 and Hyd-2 might be proton translocating systems, and this would be important for ion exchange (e.g., potassium ions), for overcoming hyper stress. In formate assays, increased V_H2_ during hypo-osmotic stress conditions was not determined. In glycerol assays, similar data were obtained as in wild type. However, in hypo-osmotic stress conditions, V_H2_ was stimulated ~50% compared to wild type.

In Hyd-2 in DCCD assays with hyper-osmotic stress conditions, V_H2_ decreased ~2.4 fold. This suggests that during hyper-osmotic stress conditions, the role of F_O_F_1_ in Hyd-2 mutant for V_H2_ increases. In glycerol assays during hypo-osmotic stress, V_H2_ is ~1.8 fold higher compared to wild type, while under hyper-osmotic stress conditions, it is similar to wild type (see Figure 1). In Hyd-3 mutant in all assays, H_2_ production was absent, which clearly shows that Hyd-3 is the main Hyd enzyme responsible for H_2_ production at pH 7.5, which is in good conformity with earlier data shown by many groups.

In Hyd-4 mutant in glucose assays, the data were similar to wild type; but when cells were subjected to hyper-osmotic stress, DCCD inhibited H_2_ production ~25% compared to the cells without DCCD inhibition (see Figure 1). In formate assays in Hyd-4 and all mutants, there are similarities with each other. Osmotic stress does not affect the H_2_ producing activity of Hyd-3, but shows that F_O_F_1_ with Hyd-1, Hyd-2 and Hyd-4 balance proton gradient across the membrane. It was experimentally shown that absence of proton ATPase affects Hyd activity, and it was suggested that F_O_F_1_ and Hyd enzyme interact to maintain proton motive force [2].

### 3.2. H_2_ production by E. coli wild type and mutant strains during hyper- and hypo-osmotic stress and inhibition by DCCD at pH 6.5

**Figure 2.**
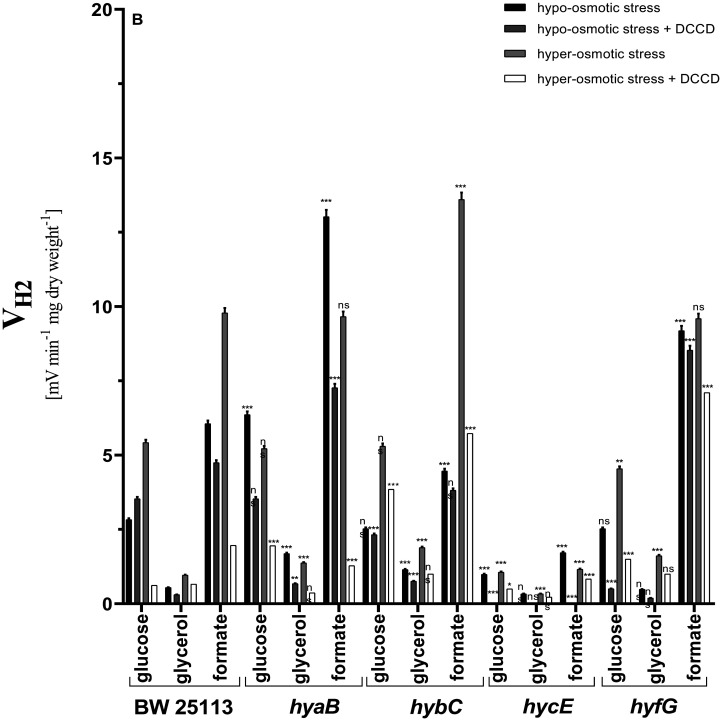
H_2_ production rates (V_H2_) by *E. coli* BW25113 wild type and different mutants with defects in Hyd enzymes under hyper- and hypo-osmotic stress during mixed carbon sources fermentation at pH 6.5. For other information, see the caption of Figure 1.

V_H2_ in wild type cells in glucose assays during hypo-osmotic conditions reached 2.8 mV E_h_/min/mg CDW, which was ~35% less than cells grown at pH 7.5 (Figure 2). Interestingly, DCCD did not inhibit H_2_ production in glucose assays, which suggests that at pH 6.5, proton ATPase and H_2_ producing Hyd enzymes (mainly Hyd-3) are not related to each other. Similar data were obtained when only glucose was fermented [8]. Under hyper-osmotic stress conditions, V_H2_ in wild type cells doubled, and DCCD totally inhibited H_2_ production. This could be because, under hyper-osmotic conditions, cells regulate proton and potassium ion gradients via metabolic cross-talk between proton ATPase and Hyd-3, responsible for H_2_ production at pH 6.5. In glycerol assays, V_H2_ similarly increased as in glucose assays.

When formate was added in the assays, V_H2_ under hypo-osmotic stress was 6 mV E_h_/min/mg CDW, but DCCD inhibited H_2_ production ~20% compared to the assays with glucose. During hyper-osmotic stress, V_H2_ increased ~1.6 fold compared to the formate assays during hypo-osmotic conditions. DCCD markedly inhibited V_H2_, which shows that during glucose or formate assays, the regulation and mechanism for surviving under hyper-osmotic stress conditions are similar. In Hyd-2 mutant in glucose assays in hyper-osmotic conditions, V_H2_ was inhibited ~25% by DCCD compared to wild type, where it was inhibited totally. The data suggest that absence of Hyd-2 might be compensated by active proton ATPase for balancing formate and H_2_ metabolism and thus proton motive force. In glycerol assays, V_H2_ reached ~1.2 mV E_h_/min/mg CDW, which is ~2.1 fold higher than in wild type under hypo-osmotic conditions. Meanwhile, DCCD inhibited V_H2_ ~35% compared to wild type, where V_H2_ was inhibited ~50% (Figure 2). The data clearly demonstrate that the role of Hyd-2 or other Hyd enzymes does not depend on glucose or glycerol as a carbon source.

V_H2_ in formate assays under hypo-osmotic conditions was similar as in wild type, but when cells were applied for hyper-osmotic stress conditions, V_H2_ increased ~3 fold. DCCD has the same effect as in wild type, suggesting that the role of proton ATPase in Hyd-2 mutant when external formate is present is similar as in wild type, in contrast to the presence of intracellular formate. Hyd-1 mutant V_H2_ in glucose assays increased ~2.2 fold compared to wild type, while DCCD inhibited V_H2_ ~50%. The data suggest that Hyd-1 at hypo-osmotic conditions works toward H_2_ uptake direction, and this process depends on proton ATPase. The data suggest that Hyd-1 at these conditions does not play a role in combating hyper-osmotic conditions. The highest V_H2_ in glycerol assays was detected in Hyd-1 mutant, reaching ~1.7 E_h_/min/mg CDW. In formate assays, V_H2_ increased ~2 fold compared to wild type, and DCCD has the same effect as in glucose assays. Under hyper-osmotic stress conditions, H_2_ production was detected to be of similar values as in wild type, which suggests that intra- or extracellular formate had no influence on working direction and role of Hyd-1.

In Hyd-3 mutant in all assays and conditions, H_2_ production was absent or residual, which clearly shows that Hyd-3 is responsible for H_2_ production. In Hyd-4 mutant, V_H2_ in glucose assays under hypo-osmotic conditions was similar as in wild type, but DCCD totally inhibited H_2_ production, in contrast to wild type cells (Figure 2). This might be because proton ATPase and Hyd-4 interact together to balance proton gradient and transfer protons to other membrane bound enzymes, and in the absence of Hyd-4, formate neutralization via Hyd-3 is disturbed, as proton transfer from proton ATPase to Hyd-3 or other systems via Hyd-4 does not take place. Under hyper-osmotic stress conditions, similar results were obtained as in wild type. In glycerol assays mainly under hyper-osmotic stress conditions, V_H2_ increased ~3 fold (Figure 2), suggesting that one of the main mechanisms that help the cell to survive under hyper-osmotic stress conditions is to neutralize protons via producing H_2_. This mechanism works in relationship with proton ATPase, which regulates overall proton motive force. Interestingly, in formate assays, V_H2_ in both conditions was similar compared to wild type. Especially, DCCD did not inhibit H_2_ production in both cases, which shows that intracellular formate disproportionation (glucose assays) and external formate neutralization mechanism are completely different, and Hyd-4 plays an important role in intracellular formate neutralization rather than extracellular.

### 3.3. H_2_ production by E. coli wild type and mutant strains during hyper- and hypo-osmotic stress and inhibition by DCCD at pH 5.5

**Figure 3.**
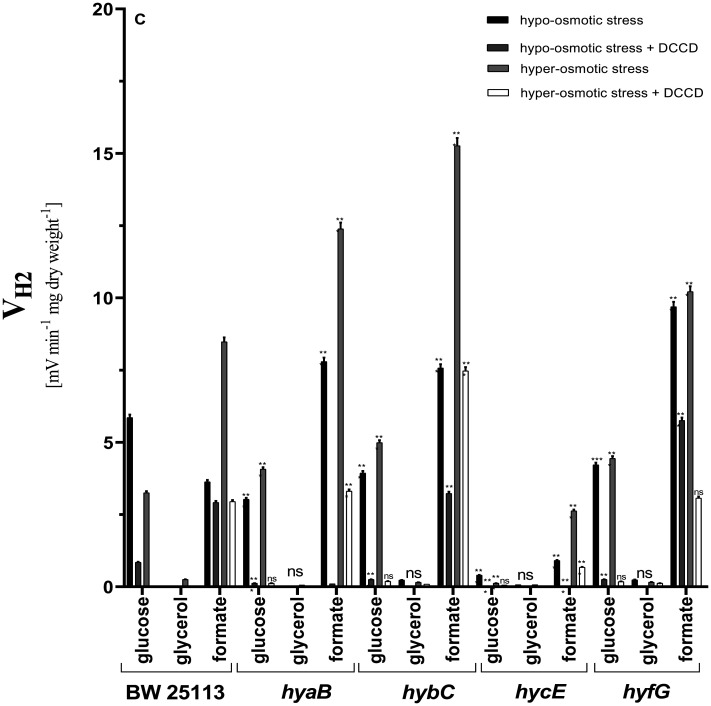
H_2_ production rates (V_H2_) by *E. coli* BW25113 wild type and different mutants with defects in Hyd enzymes under hyper- and hypo-osmotic stress during mixed carbon sources fermentation at pH 5.5. For other information, see the caption of Figure 1.

When wild type cells were subjected to hyper-osmotic stress, V_H2_, in contrast to pH 7.5, decreased ~1.8 fold, and DCCD totally inhibited H_2_ production in both conditions (Figure 3). Interestingly, in formate assays under hypo-osmotic conditions with or without DCCD, V_H2_ was similar to the results obtained for wild type grown at pH 6.5.

In all mutants in glucose assays, V_H2_ was less than in wild type, and DCCD totally inhibited H_2_ production (Figure 3). It is important to state that all Hyd enzymes are partially contributing to H_2_ production at low pH under hypo-osmotic stress conditions. In Hyd-4 mutant in formate assays, V_H2_ was higher compared to wild type and other mutants, reaching ~9.6 E_h_/min/mg CDW. However, hyper-osmotic stress conditions had no influence on V_H2_ as in wild type, which suggests that at this condition, when external formate is added, Hyd-4 is working toward the H_2_ uptake direction, and Hyd-4 is osmosensitive and participates in osmoregulation via interacting with proton ATPase. The data are supported with DCCD assays, where in Hyd-4 mutant, V_H2_ is decreased ~40%; meanwhile, under hyper-osmotic stress conditions, DCCD inhibits V_H2_ ~70% (Figure 3). In Hyd-1 mutant in formate assays, V_H2_ was ~2 fold stimulated compared to wild type, while DCCD totally inhibited H_2_ production, which suggested that proton ATPase might be involved in formate neutralization and compensate the absence of Hyd-1 for balancing the transmembrane proton gradient at low pH.

Under hyper-osmotic stress, V_H2_ was ~50% higher compared to wild type, and DCCD inhibition was similar in all mutants (Figure 3). In Hyd-2 mutant in formate assays, V_H2_ was identical as in Hyd-1 mutant. While DCCD did not totally inhibit H_2_ production, it was decreased ~50%, which suggests that at this pH, deletion of any of the Hyd enzymes is compensated by active proton ATPase for pumping protons out. Interestingly, in Hyd-1 mutant, hypo-osmotic stress conditions increased V_H2_ ~2 fold, which was similar to wild type.

## 4. Conclusions

*Escherichia coli* produces H_2_ via Hyd enzymes during mixed carbon sources (glucose and glycerol) fermentation. Overall, hyper-osmotic stress, depending on external pH, stimulated or decreased H_2_ production compared to sole carbon source fermentation, where osmotic stress inhibited H_2_ production. Particularly, at pH 7.5 in formate assays, V_H2_ was stimulated ~50% in wild type but not in mutants, while at pH 6.5 maximal stimulation was detected in *hybC* mutant. Taken together, it can be concluded that Hyd-1 and Hyd-2 contribute to osmoregulation at pH 7.5, while Hyd-4 is osmosensitive at pH 6.5 and 5.5. Contribution of proton ATPase in cell osmoregulation in metabolic crosstalk with Hyd enzymes is the main physiological phenomenon that has been suggested as depending on external pH. The data identifies the role of Hyd enzymes in cell osmoregulation and could be applied for development of enhanced H_2_ production.
